# Influenza vaccination coverage among an urban pediatric asthma population: Implications for population health

**DOI:** 10.1371/journal.pone.0269415

**Published:** 2022-10-21

**Authors:** Sarah J. Parker, Amy M. DeLaroche, Alex B. Hill, Rajan Arora, Julie Gleason-Comstock

**Affiliations:** 1 Department of Emergency Medicine, University of Michigan, Ann Arbor, MI, United States of America; 2 Division of Pediatric Emergency Medicine, Department of Pediatrics, Children’s Hospital of Michigan, Detroit, MI, United States of America; 3 Department of Urban Studies and Planning, Wayne State University, Detroit, MI, United States of America; 4 Department of Family Medicine and Public Health Sciences, School of Medicine, Wayne State University, Detroit, MI, United States of America; LSU Health Sciences Center New Orleans: Louisiana State University Health Sciences Center, UNITED STATES

## Abstract

**Introduction:**

Asthma is the most common chronic disease in children. Children with asthma are at high risk for complications from influenza; however annual influenza vaccination rates for this population are suboptimal. The overall aim of this study was to describe the characteristics of a high-risk population of children with asthma presenting to an urban pediatric emergency department according to influenza vaccination status.

**Methods:**

The study was a retrospective chart review of 4355 patients aged 2 to 18 years evaluated in a Michigan pediatric emergency department (PED) between November 1, 2017 and April 30, 2018 with an ICD-10-CM code for asthma (J45.x). Eligible patient PED records were matched with influenza vaccination records for the 2017–2018 influenza season from the Michigan Care Improvement Registry. Geospatial analysis was employed to examine the distribution of influenza vaccination status.

**Results:**

1049 patients (30.9%) with asthma seen in the PED had received an influenza vaccine. Influenza vaccination coverage varied by Census Tract, ranging from 10% to >99%. Most vaccines were administered in a primary care setting (84.3%) and were covered by public insurance (76.8%). The influenza vaccination rate was lowest for children aged 5–11 years (30.0%) and vaccination status was associated with race (p<0.001) and insurance type (p<0.001).

**Conclusions:**

Identification of neighborhood Census Tract and demographic groups with suboptimal influenza vaccination could guide development of targeted public health interventions to improve vaccination rates in high-risk patients. Given the morbidity and mortality associated with pediatric asthma, a data-driven approach may improve outcomes and reduce healthcare-associated costs for this pediatric population.

## Introduction

Asthma is the most common chronic disease in children, affecting approximately 5.5 million children under the age of eighteen in the United States [1]. Uncontrolled asthma, which affects approximately half of children with asthma [2], is associated with increased morbidity and mortality that disproportionately affects African American children and those living in poverty [3,4]. Many of these children seek care in the emergency department (ED), resulting in over half a million unscheduled ED visits per year for children under the age of 18 [5]. Given the significant health and economic burden associated with childhood asthma [5], it is a public health priority.

According to the Centers for Disease Control and Prevention (CDC) Advisory Committee on Immunization Practices (ACIP) and the American Academy of Pediatrics, children with asthma also constitute a high-risk population for complications from influenza [6,7]. Children with asthma account for over one-third of pediatric patients requiring intensive care for influenza associated complications, including pneumonia, respiratory failure, and death [8]. Therefore, vaccination is strongly recommended to help prevent severe influenza-related complications, particularly in children with asthma [9,10].

Despite ACIP recommendations, only 58% of children aged 6 months through 17 years received the influenza vaccine nationwide during the 2017–2018 season [11]. Since the 2009 influenza pandemic, the 2017–2018 influenza season was considered one of the most severe [12,13]. Trends for the 2017–2018 season showed a slight decrease in influenza vaccination coverage in younger age ranges when compared with the previous season [11]. At the state level, the Michigan Department of Health and Human Services (MDHHS) reported lower vaccination coverage in the 2017–2018 season—the influenza vaccination rate for children was only 28.6% [14]. Influenza vaccination data for children with asthma is sparse. In the 2012–2013 influenza season, 55% of children with asthma were vaccinated with only 30% having received the vaccine early in the influenza season [15]. Thus, concerted effort is needed for optimizing vaccine administration in children with asthma to reduce unexpected medically attended events.

Where a child resides plays a large role in determining their health status as it relates to racial, ethnic, cultural, and other socioeconomic factors [16,17]. In Detroit, racial health disparities are even more pronounced due to the historical and structural factors of this urban population: an estimated 83% of individuals are African American and 40% of residents live below the poverty line [18]. African American children are 2.5 times more likely to visit the ED for asthma and the prevalence of persistent asthma is 33% higher than in white children [18]. Among this higher-risk group of children with asthma, racial disparities and cultural differences in vaccine acceptance may mitigate vaccination coverage [19,20], but the vaccination rate for this specific population is unknown. Understanding the geospatial distribution of vaccine uptake may provide important information on where to direct intervention efforts to improve vaccine uptake.

In this collaborative research study, we analyze medical record data of patients presenting to an urban pediatric emergency department (PED) with a history of asthma or for asthma-related symptoms according to influenza vaccination status during the 2017–2018 influenza season. We describe demographic and clinical characteristics, assess differences in asthma- and influenza-associated characteristics according to patient age, and examine geospatial associations in influenza vaccination among this urban population.

## Methods

### Study setting, design, and participants

This retrospective study was conducted at an urban free-standing children’s hospital located in Southeast Michigan with a Level 1 trauma center and over 85,000 PED visits annually. Records of patients aged 2 to 18 years evaluated in the PED between November 1, 2017 and April 30, 2018 and assigned an International Classification of Diseases, 10th Revision, Clinical Modification (ICD-10-CM) diagnosis code for asthma (J45.x) at PED disposition were identified using Arbor Metrix. Arbor Metrix is an electronic data repository for the Michigan Emergency Department Improvement Collaborative (MEDIC) [21]. The study dates were selected to reflect the peak influenza period for the 2017–2018 influenza season [22,23]. In order to include all children with asthma seeking PED care, all visits for patients with an ICD-10-CM code for asthma as the primary diagnosis or secondary diagnoses were included to reflect either an acute visit for asthma or a history of asthma [24]. To avoid including patients with a history of viral-induced wheeze, children < 2 years of age were excluded. Patients presenting to an affiliated satellite suburban PED or who resided outside of Michigan based on the registered home address in the electronic medical record (EMR) were also excluded.

Administrative permission to access the raw data was received from MEDIC and permission to access identified patient records in the Michigan Care Improvement Registry (MCIR) was received from the Michigan Department of Health and Human Services (MDHHS) (data available upon request and registry permission, see Declarations). The Institutional Review Boards of Wayne State University (111218MP2E) and the MDHHS (201901-05-EA) reviewed and approved the conduct of this study.

### Data collection, variables, and definitions

Data obtained from MEDIC for eligible patients included demographic (age, gender), administrative (date of PED visit), and clinical (PED disposition) variables. The EMR was reviewed by trained study personnel using a standardized data collection tool to abstract additional variables including: patient race or ethnicity, primary insurance type, home address, whether the patient presented for asthma symptoms (based on the treating PED clinician’s diagnosis), whether the patient was diagnosed with influenza (based on the treating PED clinician’s diagnosis), and whether influenza testing and results were documented. Influenza testing results were recorded if they were available in the EMR at any time during the patient’s visit, such as testing obtained in the PED directly, documented from a prior clinic or ED visit, or performed during inpatient hospital admission.

Identified patient records were linked to the MCIR to determine whether the patient had received an influenza vaccination at any point during the 2017–2018 season from September 1, 2017-April 30, 2018. MCIR collects immunization information from statewide providers and collates the information into a comprehensive immunization record-keeping system [25]. Date of vaccine administration, type of facility administering vaccine, and funding eligibility of vaccine administration were noted. Patients with no matching MCIR record were subsequently removed from the analysis because it was not possible to assess the vaccination status of these patients.

As children less than 5 years of age are especially high risk for influenza complications [26], ages were grouped into the following ranges: 2–4 years (preschool), 5–11 years (school-aged), and 12–18 years (adolescence). Vaccine administration date was classified as “early” or “late” according to CDC guidelines, with “early” vaccination defined as influenza vaccine administered by October 31, 2017 [27]. For the purposes of this research, patient home addresses were initially grouped into three Statewide regions (Southeast, Central, and Western) according to county [28]. City of Detroit ZIP codes were categorized according to five neighborhood regions (East Riverfront, Northeast, Southwest, Westside, and Woodward Corridor) [29]. Data were aggregated and analyzed geospatially at the 2019 Census Tract boundary level within the City of Detroit. In health research, Census Tracts are regularly used as a default for neighborhood [30]. Data were limited to Census Tracts in the City of Detroit due to the high level of patient density allowing for the low likelihood of identifying individual patient locations.

### Data analysis

General frequency and central tendency measures were performed to summarize descriptive statistics for patient characteristics. Statistical hypothesis tests were assessed for statistical significance using chi-square tests at a two-tailed alpha of 0.05. This test statistic was used to assess independence between vaccination status or age group and discrete factors. Analyses were performed using IBM SPSS Statistics, Version 25.0 (Armonk, NY: IBM Corp.). Hotspot analyses were run using Global Moran’s I statistic and the Local Indicators of Spatial Autocorrelation (LISA) with GeoDa 1.18 software [31]. Moran’s I and LISA were run in univariate hotspot analyses for vaccinated and unvaccinated patients as well as bivariate analyses accounting for socio-demographic characteristics of patients.

The Global Moran’s I statistic for spatial autocorrelation was used to assess the correlation among neighboring observations and to identify patterns of spatial clustering [32]. The spatial weights were row-standardized and a spatial weight matrix was constructed that contained information on the demographic details for each Census Tract, including the Census Tract itself. Non-neighboring Census Tracts were assigned a weight of zero. Moran’s I values range from -1 (dispersed) to +1 (clustered). A Moran’s I value of 0 suggests complete spatial randomness. A random permutation procedure recalculates a statistic many times by reshuffling the data values among the Census Tracts to generate a reference distribution. The calculated statistic, based on the observed spatial pattern, is then compared to the reference distribution, and a pseudo significance level (pseudo *p* value) is computed. LISA provides information related to the location of spatial clusters and outliers and the types of spatial correlation. Local statistics are important because the magnitude of the spatial autocorrelation was not necessarily uniform over the study area [33,34]. This analysis used 999 permutations to determine the differences among the spatial units. A positive value for the local Moran’s I index indicates that a feature has neighboring features that have similarly high or low attribute values, meaning that it is a part of a cluster. A negative value indicates that a feature has neighboring features that have dissimilar values, indicating that it is an outlier. In either circumstance, the *p* value for the feature must be small enough for the cluster or outlier to be considered statistically significant. LISA enables distinctions to be made among a statistically significant (0.05 level) cluster of high values (HH), a cluster of low values (LL), an outlier in which a high value is surrounded mostly by low values (HL), and an outlier in which a low value is surrounded mostly by high values (LH).

## Results

The MEDIC data repository identified 4355 patient visits to the PED between November 1, 2017 and April 30, 2018. A total of 181 visits were excluded for the following reasons: presented to a satellite suburban PED, 44; resided out-of-state, 12; no matching MCIR record, 125. Among 4174 eligible patient visits, 3392 unique patients were identified and 1049 / 3392 (30.9%) received an influenza vaccine during the study period. Overall, the majority of PED visits were by African Americans (80.7%), males (57.8%), children under 8 years of age (54.8%), and children with public or no insurance (83.0%). The median patient age for vaccinated and unvaccinated groups was 7 years (IQR 4.0–11.0). Overall, 23.9% of PED visits were associated with influenza testing. Of those with influenza testing, a positive influenza test result was documented at the time of the visit in 22.9% of encounters, with 83.4% of positive results due to influenza A. Additional demographic and clinical characteristics by vaccination status for the PED visit are reported in Table 1.

**Table 1:** Study population demographics and clinical characteristics according to vaccination status.

	Overall n = 4174 n (%)	Vaccinated n = 1323 n (%)	Unvaccinated n = 2851 n (%)	p-value
Gender Female Male	1761 (42.2) 2413 (57.8)	548 (41.4) 775 (58.6)	1213 (42.5) 1638 (57.5)	0.493
Age, years 2–4 5–11 12–18	1239 (29.7) 2008 (48.1) 927 (22.2)	425 (32.1) 602 (45.5) 296 (22.4)	814 (28.6) 1406 (49.3) 631 (22.1)	0.036
Race American Indian/Alaska Native Asian Black/African American White Patient declined or unknown	3 (0.1) 12 (0.3) 3369 (80.7) 273 (6.5) 517 (12.4)	3 (0.2) 6 (0.5) 970 (73.3) 109 (8.2) 235 (17.8)	0 6 (0.2) 2399 (84.1) 164 (5.8) 282 (9.9)	<0.001
Ethnicity Hispanic/Latino Not Hispanic/Latino	179 (4.3) 3995 (95.7)	81 (6.1) 1242 (93.9)	98 (3.4) 2753 (96.6)	<0.001
Detroit Neighborhood (n = 3069) East Riverfront Northeast Southwest Westside Woodward Corridor	294 (9.6) 803 (26.2) 319 (10.4) 1222 (39.8) 431 (14.0)	98 (10.5) 251 (27.0) 122 (13.1) 335 (36.0) 124 (13.3)	196 (9.2) 552 (25.8) 197 (9.2) 887 (41.5) 307 (14.4)	0.002
Payer Type Private Public No insurance	711 (17.0) 3306 (79.2) 157 (3.8)	247 (18.7) 1047 (79.1) 29 (2.2)	464 (16.3) 2259 (79.2) 128 (4.5)	<0.001
PED Disposition Admission* Discharge Other	1066 (25.5) 3067 (73.5) 41 (1.0)	364 (27.5) 943 (71.3) 16 (1.2)	702 (24.6) 2124 (74.5) 25 (0.9)	0.072
Presenting for Asthma Symptoms Yes No	1753 (42.0) 2421 (58.0)	532 (40.2) 791 (59.8)	1221 (42.8) 1630 (57.2)	0.111
Influenza Test Documented Yes No	999 (23.9) 3175 (76.1)	333 (25.2) 990 (74.8)	666 (23.4) 2185 (76.6)	0.202
Influenza Test Result (n = 999) Positive Negative Unknown	229 (22.9) 767 (76.8) 3 (0.3)	65 (19.5) 266 (79.9) 2 (0.6)	164 (24.6) 501 (75.2) 1 (0.2)	0.098
Influenza Type (n = 229) Influenza A positive Influenza B positive	191 (83.4) 38 (16.6)	56 (86.2) 9 (13.8)	135 (82.3) 29 (17.7)	0.482

*Includes observation and inpatient hospital admissions.

The majority of PED visits were from patients residing in the Southeast region of the state (4161, 99.7%). Thirteen visits (0.3%) were from children residing outside of the Southeast region. Most patients residing in the Southeast region of the state were living in the city of Detroit (2482 / 3392 patients, 73.2%). Within the city of Detroit, the relative majority of patients resided within the Westside neighborhood (40.0%), followed by Northeast (26.5%), Woodward Corridor (14.1%), Southwest (10.7%), and East Riverfront (8.7%). Geospatial analysis was conducted by Census Tract for these five neighborhood regions. As shown in Table 1, vaccination status varied by neighborhood: the greatest proportion of unvaccinated visits were by patients who lived in the Westside (41.5%). Within each neighborhood, the highest proportion of vaccinated patients lived in the Southwest (100 / 266, 37.6%), followed by East Riverfront (66 / 216, 30.6%), Northeast (201 / 658. 30.5%), Woodward Corridor (100 / 349, 28.7%), and Westside (270 / 993, 27.2%).

Among vaccinated patients (Table 2, n = 1049), there were no notable differences in the timing of the vaccination with 49.1% receiving an “early” vaccine and 50.9% receiving a “late” vaccine. Most vaccines were administered in a primary care setting (84.3%) and through publicly funded campaigns (76.8%).

**Table 2:** Vaccine administration characteristics for vaccinated children, n (%) (n = 1049).

Vaccine administration date* Early Late	515 (49.1) 534 (50.9)
Location administering vaccine Primary Care Subspecialty or Other Clinic± Hospital Local Health Department Pharmacy	884 (84.3) 81 (7.7) 42 (4.0) 23 (2.2) 19 (1.8)
Vaccine administration publicly funded Yes No	806 (76.8) 243 (23.2)

*Early vaccine administration date (September-October) and late vaccine administration date (November-April) determined according to CDC recommendations [27].

±Includes vaccines administered at the following locations: Correctional clinic, Local health department satellite clinic, School-based health center, Specialty clinic, Teen health center.

Although many visits captured patients with a history of asthma (58.0%), a greater proportion of unvaccinated patients were presenting to the PED for asthma-related symptoms (42.8% vs. 40.2%) and tested positive for influenza (24.6% vs. 19.5%). Among patients presenting to the PED for asthma related symptoms (n = 1753, 42.0%), only 30.3% (p-value 0.111) had received an influenza vaccination for the 2017–2018 season. When stratified by age (Table 3), the proportion of PED visits for an acute asthma exacerbation decreased with age, from 55.5% of visits among children aged 2–4 years to 24.9% of visits among children aged 12–18 years. Notably, a smaller proportion of visits for ages 5–11 (30.0%) were by children who had received the influenza vaccine. Over half of visits across all age groups were by children with late vaccine administration.

**Table 3:** Asthma and influenza characteristics by patient age groups.

	Age in years			p-value
	2–4	5–11	12–18	
Presenting for asthma exacerbation Yes No	688 (55.5) 551 (44.5)	834 (41.5) 1174 (58.5)	231 (24.9) 696 (75.1)	<0.001
Influenza case Yes No	80 (6.5) 1159 (93.5)	127 (6.3) 1881 (93.7)	28 (3.0) 899 (97.0)	<0.001
Influenza test documented Yes No	406 (32.8) 833 (67.2)	453 (22.6) 1555 (77.4)	140 (15.1) 787 (84.9)	<0.001
Influenza test result (n = 999) Positive Negative Unknown	75 (18.5) 329 (81.0) 2 (0.5)	126 (27.8) 326 (72.0) 1 (0.2)	28 (20.0) 112 (80.0) 0	0.015
Vaccinated Yes No	425 (34.3) 814 (65.7)	602 (30.0) 1406 (70.0)	296 (31.9) 631 (68.1)	0.036
Vaccine administration date* (n = 1049) Early Late	166 (49.6) 169 (50.4)	229 (48.9) 239 (51.1)	120 (48.8) 126 (51.2)	0.979

Reported by overall patient visits to the pediatric emergency department (PED) (n = 4174) unless otherwise noted.

*Early vaccine administration date (September-October) and late vaccine administration date (November-April) determined according to CDC recommendations [27].

Additional analyses were performed by gender, race, and primary payer type. None of the analyses by gender were statistically significant. Conversely, whether the patient presented to the PED for asthma related symptoms (p-value <0.001), was diagnosed with influenza (p-value 0.023), received an influenza vaccination (p-value <0.001), or received an “early” influenza vaccine (p-value 0.008) were all statistically significant by racial group. Among PED visits for patients of African American race, a greater proportion presented to the PED for asthma-related symptoms (44.3%), were diagnosed with influenza (5.3%), were unvaccinated (71.2%), and received a “late” influenza vaccination (54.5%) than non-Hispanic white patients. Although not statistically significant (p-value 0.053), a smaller proportion of PED visits for African American patients were associated with influenza testing (23.2%). Finally, by primary payer type, both influenza testing documented at the time of the PED visit (p-value 0.021) and vaccination status (<0.001) were statistically significant. Although only a minority of PED visits were associated with influenza testing, 14.6% of those with no insurance had influenza testing documented in contrast to 24.6% of those with private insurance and 24.2% of those with public insurance. A greater proportion of visits with private insurance were associated with vaccination (34.7%) in contrast to visits with public (31.7%) or no insurance (18.5%).

**Fig 1:**
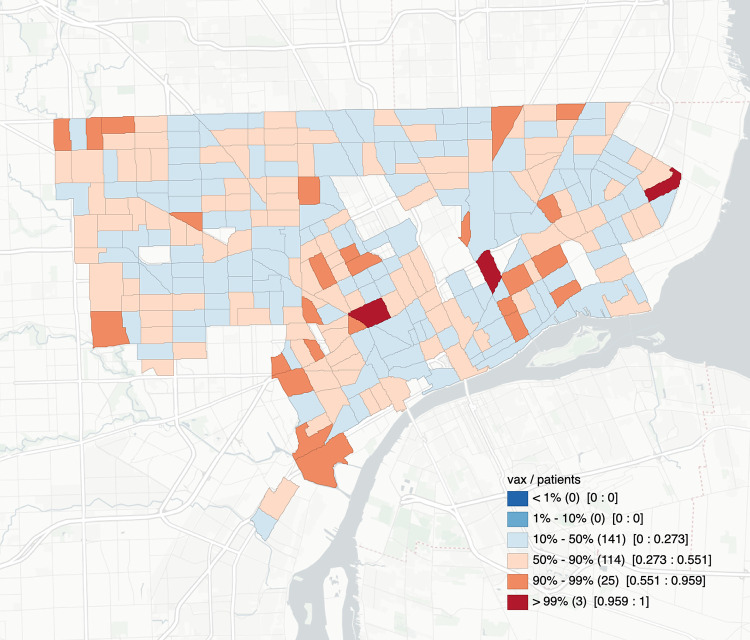
Influenza vaccination coverage percentiles by Census Tract in Detroit.

**Fig 2:**
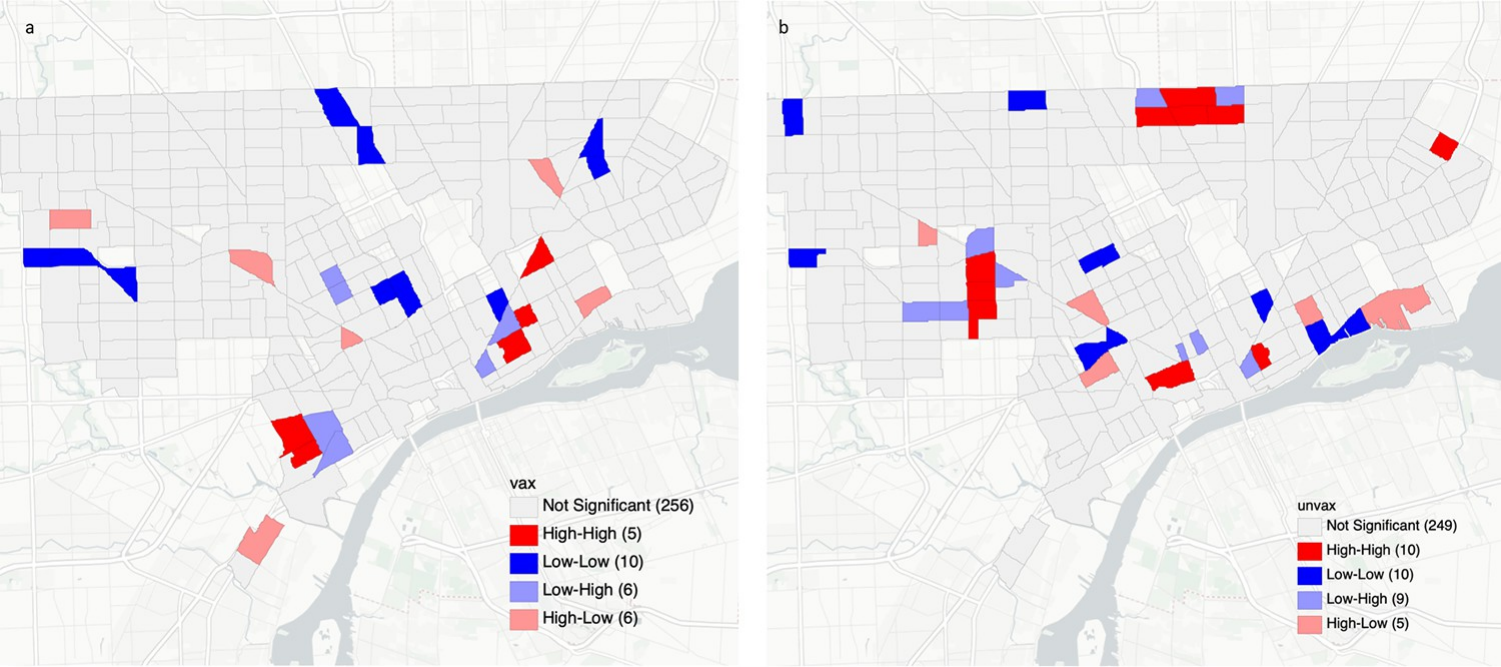
Hotspot clusters of vaccinated patients (a) and unvaccinated patients (b). Clusters are categorized as high-high cluster (red), low-low cluster (blue), low-high outlier (light blue), and high-low outlier (light red).

We conducted spatial analyses at the Census Tract level to explore levels of clustering associated with vaccination status. Fig 1 displays the raw rate of influenza vaccination coverage by percentile by Census Tract in Detroit. Vaccination coverage among patients across Detroit ranged from a low of 10% to 100% in Census Tracts across the city. Vaccination coverage of <50% is particularly notable in the areas denoted in blue on Fig 1. There were significant degrees of clustering among vaccinated (Fig 2A) and unvaccinated (Fig 2B) patients with unvaccinated patients generating the greatest number of hotspots in the city.

**Fig 3:**
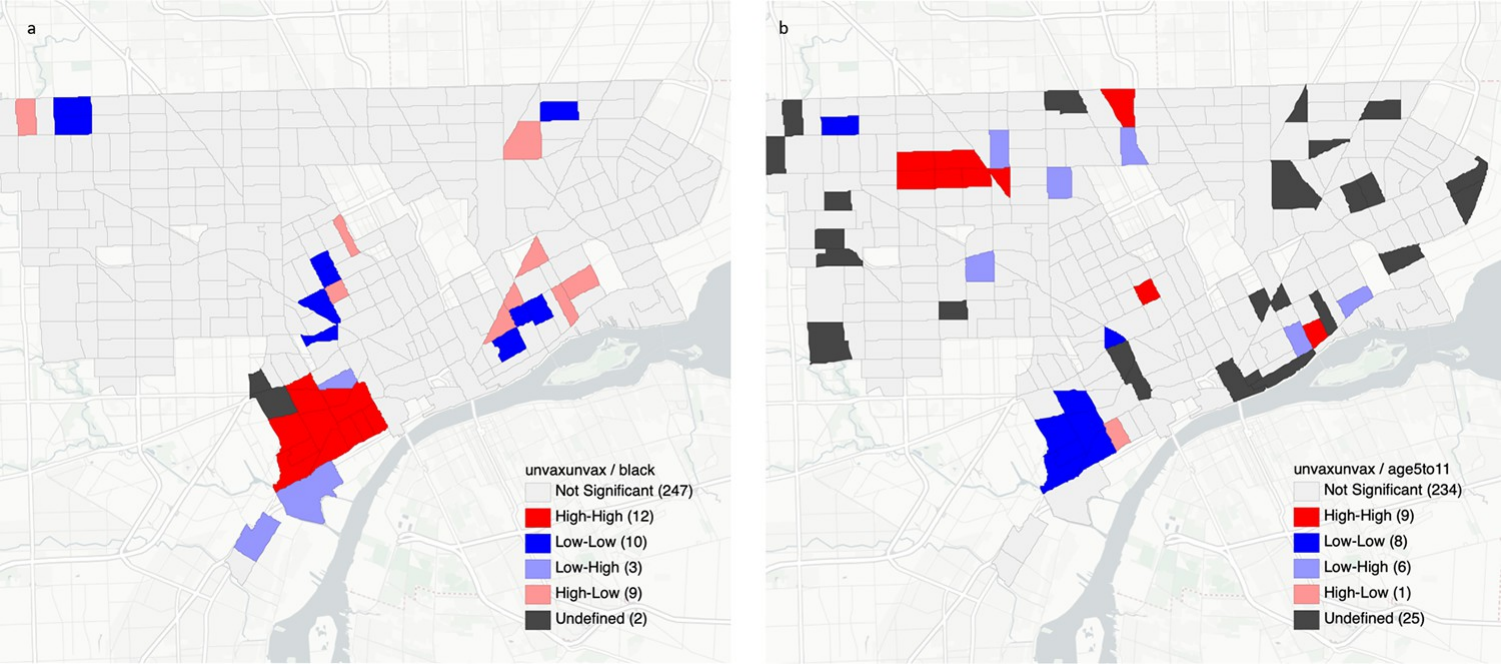
Hotspot clusters of unvaccinated patients who were Black or African American (a) or age 5–11 years (b). Clusters are categorized as high-high cluster (red), low-low cluster (blue), low-high outlier (light blue), and high-low outlier (light red).

The Global Moran’s I statistic (Table 4) identified two significant findings for positive spatial autocorrelation among unvaccinated patients: Black or African American race (Moran’s I 0.080; p-value 0.014) and being in the age 5 to 11 group (Moran’s I 0.070; p-value 0.017). Our significant geospatial findings suggest that clustering patterns are not likely to be the result of random chance. These two significant findings were also examined using LISA to pinpoint hotspots (Fig 3).

**Table 4:** Results of statistical analyses of Global Moran’s I measuring spatial autocorrelation.

	Vaccinated		Unvaccinated	
	Moran’s I	p-value*	Moran’s I	p-value*
Flu Case	-0.015	0.306	-0.002	0.496
Asthma Exacerbation	0.024	0.178	.052	0.046
White	0.048	0.041	-0.034	0.078
Black	-0.029	0.169	**0.080**	**0.014**
Ages 2 to 4	-0.009	0.405	0.028	0.151
Ages 5 to 11	0.039	0.094	**0.070**	**0.017**
Ages 12 to 18	0.019	0.219	0.031	0.124

Significant findings are noted in bold.

*Indicates a pseudo p-value at permutation of 999.

## Discussion

Vaccine administration is recommended to prevent severe influenza-related complications in pediatric patients with asthma. This study suggests suboptimal vaccination coverage, as seen during the 2017–2018 season, when less than one-third of asthma patients presenting to the PED had received an influenza vaccine. This is similar to the rate seen in another study in a minority population [35]. While influenza vaccination efforts may be hampered by factors such as age, race, and insurance status, our results highlight that vaccination rates vary substantially with Census Tracts. Geospatial analysis can offer information that public health professionals can use to develop initiatives that will promote vaccine acceptance rates in specific higher risk communities.

Few previous studies have reported influenza vaccination rates for pediatric asthma patients, with reported vaccination coverage much higher than found in this research [15,36]. While our findings illustrate the limitations of applying national estimates to specific high-risk populations, both our data and national surveys indicate that only one-third of patients with asthma are vaccinated “early” (i.e., by end of October) in the influenza season. Earlier starts to the influenza season, as during 2017–2018, further underscore the importance of advocating for early vaccination. Vaccination does not immediately offer protection from influenza to a child [37]. Thus, to have the greatest benefit, it is important for vaccination to occur well before there is widespread influenza activity in the community. More than 50 percent of vaccinated children in our population received the influenza vaccine beyond recommended administration dates. Effective education and communication on the importance of receiving an influenza vaccine early in the season is merited and may be lacking for this high-risk population [38]. Amid the current pandemic of coronavirus disease 2019, interactions advocating for influenza vaccination may be increasingly important [39], especially in pediatric groups where disparities in vaccination coverage are already known [40].

The “Improving Childhood Influenza Immunization: A Five Year Progress Report” published by the National Foundation for Infectious Diseases highlights action items for improving influenza vaccination coverage, including: provider education and recommendation for vaccination, providing tailored vaccine communication to address community needs, expanding vaccination opportunities to non-traditional settings, and encouraging vaccination to all members of the family [41]. Schools are a key setting where some of these strategies may be addressed; yet, the National Heart, Lung, and Blood Institute guide “Managing Asthma: A Guide for Schools” does not explicitly recommend that schools advocate for influenza vaccination [42]. School-based programs, including a student curriculum and a parental brochure, have been shown to be effective at shifting attitudes and motivating participants to receive recommended vaccines [43]. Additionally, prior to the start of influenza season, incorporation of influenza vaccine education into existing virtual [44] or school-based programs, such as the School-based Asthma, Allergy & Anaphylaxis Management Program (SA^3^MPRO^TM^) and Step-Up Asthma, may be a beneficial approach for improving vaccine uptake [45–47]. Efforts can also be focused on children lacking insurance who may not be aware of eligibility to receive vaccines through programs such as Vaccines for Children [48]. Collectively, these interventions may be most impactful when delivered in schools or community health centers located in neighborhoods we identified with low vaccination rates.

While primary care locations played a significant role in vaccinating children in this study, most patients in this population had not received the influenza vaccine. To ensure that any encounter with a healthcare professional during the influenza season is viewed as an opportunity to vaccinate, approaches to vaccination both inside and outside of the primary care setting (i.e., “non-traditional” options) should be considered [49]. Non-traditional options are particularly important in an urban setting where many children do not have an established relationship with a primary care provider [50–52]. Furthermore, a recent systematic review highlighted transportation as a common issue with accessing care but noted that transportation-only interventions may not be sufficient for improving health outcomes [53]. As improved health care access has been positively associated with influenza vaccination coverage, the opportunity to improve vaccine administration in non-traditional settings–such as at the hospital, pharmacy, or mobile health unit–may be an appealing alternative in an urban population [54–56]. Paired with education and communication interventions in schools and community health centers, “non-traditional” healthcare interactions serve as an added piece for building “reminder systems” in urban settings [35].

Approximately one-quarter of hospitalized children in this study were unvaccinated. Hospitalization is an additional opportunity to vaccinate. One study from a large children’s hospital in Texas reported successfully improving vaccination rates among admitted children with asthma from 13.3% to 57.4% [57]. In addition to inpatient initiatives, hospitals can also consider immunization centers, which, depending upon the needs of the community, may be tailored to non-traditional hours to further improve access [58]. Outside of the hospital setting, only a minority of children in our study were vaccinated in a pharmacy. Underutilization of neighborhood pharmacies represents a missed opportunity to improve vaccination capacity as pharmacies have proven that they can play an integral role in community immunization initiatives [59]. Finally, incorporating influenza vaccination into existing mobile health units may be an efficient way to target neighborhoods we identified with lower rates of vaccination while eliminating transportation barriers to care.

### Limitations

The retrospective nature of this study may undermine the accuracy of the data as analyses were limited by the information available in the EMR. Second, the context may be unique, and the findings noted herein may not be generalizable to other settings, i.e., different racial communities or non-urban areas and healthcare environments. Third, it is unknown whether the vaccination status of the patient correlated with the acuity of the PED visit, or the clinical context of care received during the PED visit. Fourth, study findings are limited by insufficient information to determine whether the influenza vaccination for the 2017–2018 season was complete for any given individual. Records were not available from prior seasons to account for those patients who may have been receiving the vaccination for the first time as these patients would have required two doses during the included influenza season to be considered completely vaccinated. Finally, there could be other factors not measured in this research that would explain the observed geospatial patterns.

## Conclusions

Influenza vaccination among pediatric patients with asthma is suboptimal. Geospatial analysis is an important population health tool that can help highlight communities with a greater need for targeted public health interventions, which can further enable engagement of families, schools, hospitals, and health care providers, including pharmacies, within that neighborhood. More studies are needed to determine the efficacy of this data-driven approach to public health in improving influenza vaccine uptake in children with asthma. Findings from this study may be used by public health professionals to inform community level interventions to educate on the importance of vaccination timing, implement reminder systems, and improve vaccination rates in preparation for future influenza and other viral seasons.

## Abbreviations

ED: Emergency Department
PED: Pediatric Emergency Department
CDC: Centers for Disease Control and Prevention
ACIP: Advisory Committee on Immunization Practices
MDHHS: Michigan Department of Health & Human Services
MEDIC: Michigan Emergency Department Improvement Collaborative
EMR: Electronic Medical Record
ICD-10-CM: International Classification of Diseases, 10th Revision, Clinical Modification
MCIR: Michigan Care Improvement Registry
LISA: Local Indicators of Spatial Autocorrelation

## References

[1] Most Recent National Asthma Data [Internet]. Centers for Disease Control and Prevention; [updated May 25, 2022. Available from: https://www.cdc.gov/asthma/most_recent_national_asthma_data.htm.

[2] Uncontrolled Asthma Among Children With Current Asthma, 2018–2020 [Internet]. Centers for Disease Control and Prevention; [updated July 1, 2022. Available from: https://www.cdc.gov/asthma/asthma_stats/uncontrolled-asthma-children-2018-2020.htm.

[3] Asthma and African Americans: U.S. Department of Health and Human Services Office of Minority Health; [updated February 11, 2021. Available from: https://minorityhealth.hhs.gov/omh/browse.aspx?lvl=4&lvlid=15.

[4] Akinbami LJ, Simon AE, Rossen LM. Changing Trends in Asthma Prevalence Among Children. Pediatrics. 2016;137(1):1–7. doi: 10.1542/peds.2015-2354 26712860 PMC4755484

[5] Perry R, Braileanu G, Palmer T, Stevens P. The Economic Burden of Pediatric Asthma in the United States: Literature Review of Current Evidence. Pharmacoeconomics. 2019;37(2):155–67. doi: 10.1007/s40273-018-0726-2 30315512 PMC6386052

[6] Grohskopf LA, Alyanak E, Ferdinands JM, Broder KR, Blanton LH, Talbot HK, et al. Prevention and Control of Seasonal Influenza with Vaccines: Recommendations of the Advisory Committee on Immunization Practices, United States, 2021–22 Influenza Season. MMWR Recomm Rep. 2021;70(5):1–28. doi: 10.15585/mmwr.rr7005a1 34448800 PMC8407757

[7] Recommendations for Prevention and Control of Influenza in Children, 2021–2022. Pediatrics. 2021;148(4). 10.1542/peds.2021-053745 34493538

[8] Dawood FS, Kamimoto L, D’Mello TA, Reingold A, Gershman K, Meek J, et al. Children with asthma hospitalized with seasonal or pandemic influenza, 2003–2009. Pediatrics. 2011;128(1):e27–32. doi: 10.1542/peds.2010-3343 21646257

[9] Miller EK, Griffin MR, Edwards KM, Weinberg GA, Szilagyi PG, Staat MA, et al. Influenza burden for children with asthma. Pediatrics. 2008;121(1):1–8. doi: 10.1542/peds.2007-1053 18166550

[10] Prevention and control of seasonal influenza with vaccines. Recommendations of the Advisory Committee on Immunization Practices—United States, 2013–2014. MMWR Recomm Rep. 2013;62(Rr-07):1–43. 24048214

[11] Estimates of Flu Vaccination Coverage among Children [Internet]. Centers for Disease Control and Prevention; [updated January 18, 2022. Available from: https://www.cdc.gov/flu/fluvaxview/coverage-1718estimates-children.htm.

[12] 2017–2018 Estimated Influenza Illnesses, Medical visits, Hospitalizations, and Deaths and Estimated Influenza Illnesses, Medical visits, Hospitalizations, and Deaths Averted by Vaccination in the United States [Internet]. Centers for Disease Control and Prevention; [updated July 22, 2022. Available from: https://www.cdc.gov/flu/about/burden-averted/2017-2018.htm.

[13] Past Seasons Estimated Influenza Disease Burden [Internet]. Centers for Disease Control and Prevention; [updated October 1, 2020. Available from: https://www.cdc.gov/flu/about/burden/past-seasons.html.

[14] County Immunization Report Cards [Internet]. Michigan Department of Health & Human Services; [Available from: https://www.michigan.gov/mdhhs/adult-child-serv/childrenfamilies/immunization/localhealthdepartment/county-immunization-report-card.

[15] Simon AE, Ahrens KA, Akinbami LJ. Influenza Vaccination Among US Children With Asthma, 2005–2013. Acad Pediatr. 2016;16(1):68–74. doi: 10.1016/j.acap.2015.10.006 26518382 PMC4739299

[16] Purnell TS, Calhoun EA, Golden SH, Halladay JR, Krok-Schoen JL, Appelhans BM, et al. Achieving Health Equity: Closing The Gaps In Health Care Disparities, Interventions, And Research. Health Aff (Millwood). 2016;35(8):1410–5. doi: 10.1377/hlthaff.2016.0158 27503965

[17] Poowuttikul P, Saini S, Seth D. Inner-City Asthma in Children. Clin Rev Allergy Immunol. 2019;56(2):248–68. doi: 10.1007/s12016-019-08728-x 30666508

[18] DeGuire P, Cao B, Wisnieski L, Strane D, Wahl R, Lyon-Callo S, et al. Detroit: The Current Status of the Asthma Burden. Michigan Department of Health and Human Services Bureau of Disease Control, Prevention and Epidemiology; 2016.

[19] Santibanez TA, Kennedy ED. Reasons given for not receiving an influenza vaccination, 2011–12 influenza season, United States. Vaccine. 2016;34(24):2671–8. 27118168 10.1016/j.vaccine.2016.04.039 PMC5751433

[20] Bernstein HH, Bocchini JA Jr. The Need to Optimize Adolescent Immunization. Pediatrics. 2017;139(3). doi: 10.1542/peds.2016-4186 28167517

[21] Kocher KE, Arora R, Bassin BS, Benjamin LS, Bolton M, Dennis BJ, et al. Baseline Performance of Real-World Clinical Practice Within a Statewide Emergency Medicine Quality Network: The Michigan Emergency Department Improvement Collaborative (MEDIC). Ann Emerg Med. 2020;75(2):192–205. doi: 10.1016/j.annemergmed.2019.04.033 31256906

[22] Summary of the 2017–2018 Influenza Season [Internet]. Centers for Disease Control and Prevention; [updated September 5, 2019. Available from: https://www.cdc.gov/flu/about/season/flu-season-2017-2018.htm.

[23] Weekly US Map: Influenza Summary Update [Internet]. Centers for Disease Control and Prevention; [updated August 19, 2022. Available from: https://www.cdc.gov/flu/weekly/usmap.htm.

[24] ICD-10-CM Code for Asthma J45 [Internet]. Codify by AAPC; 2022 [Available from: https://www.aapc.com/codes/icd-10-codes/J45.

[25] MCIR–Michigan Care Improvement Registry [Internet]. Michigan Public Health Institute; [updated March 2022. Available from: https://www.mcir.org/.

[26] Flu & Young Children [Internet]. Centers for Disease Control and Prevention; [updated October 25, 2021. Available from: https://www.cdc.gov/flu/highrisk/children.htm.

[27] Influenza Vaccination: A Summary for Clinicians [Internet]. Centers for Disease Prevention and Control; [updated July 14, 2022. Available from: https://www.cdc.gov/flu/professionals/vaccination/vax-summary.htm.

[28] MI Safe Start Map [Internet]. Ann Arbor: University of Michigan; [updated December 13, 2021. Available from: https://www.mistartmap.info/cdc-indicators.

[29] Goodman L, Bai B. New data show continued but uneven recovery in Detroit’s housing market [Internet]. 2016 [Available from: https://www.urban.org/urban-wire/new-data-show-continued-uneven-recovery-detroits-housing-market.

[30] Coulton CJ, Jennings MZ, Chan T. How big is my neighborhood? Individual and contextual effects on perceptions of neighborhood scale. Am J Community Psychol. 2013;51(1–2):140–50. doi: 10.1007/s10464-012-9550-6 22886284

[31] Science TCfSD. GeoDa: An Introduction to Spatial Data Analysis [Internet]. Chicago: The University of Chicago; 2022 [Available from: https://spatial.uchicago.edu/geoda.

[32] Boots BN, Getis A. Point Pattern Analysis: Books on Demand; 1988.

[33] Anselin L. Local Indicators of Spatial Association—LISA. Geographical Analysis. 1995;27(2):93–115.

[34] Ord JK, Getis A. Local Spatial Autocorrelation Statistics: Distributional Issues and an Application. Geographical Analysis. 1995;27(4):286–306.

[35] Uwemedimo OT, Findley SE, Andres R, Irigoyen M, Stockwell MS. Determinants of influenza vaccination among young children in an inner-city community. J Community Health. 2012;37(3):663–72. doi: 10.1007/s10900-011-9497-9 22045471

[36] Flu Vaccination among Children with Current Asthma [Internet]. Centers for Disease Control and Prevention; [updated December 29, 2017. Available from: https://www.cdc.gov/asthma/asthma_stats/flu_vaccination_child.html.

[37] Key Facts About Seasonal Flu Vaccine [Internet]. Centers for Disease Control and Prevention; [updated July 14, 2022. Available from: https://www.cdc.gov/flu/prevent/keyfacts.htm.

[38] Hofstetter AM, LaRussa P, Rosenthal SL. Vaccination of adolescents with chronic medical conditions: Special considerations and strategies for enhancing uptake. Hum Vaccin Immunother. 2015;11(11):2571–81. doi: 10.1080/21645515.2015.1067350 26212313 PMC4685675

[39] Yang MJ, Rooks BJ, Le TT, Santiago IO, 3rd, Diamond J, Dorsey NL, et al. Influenza Vaccination and Hospitalizations Among COVID-19 Infected Adults. J Am Board Fam Med. 2021;34(Suppl):S179–s82. doi: 10.3122/jabfm.2021.S1.200528 33622834

[40] DeSilva MB, Haapala J, Vazquez-Benitez G, Daley MF, Nordin JD, Klein NP, et al. Association of the COVID-19 Pandemic With Routine Childhood Vaccination Rates and Proportion Up to Date With Vaccinations Across 8 US Health Systems in the Vaccine Safety Datalink. JAMA Pediatr. 2022;176(1):68–77. 34617975 10.1001/jamapediatrics.2021.4251 PMC8498937

[41] Coalition CII. Improving Childhood Influenza Immunization: A Five Year Progress Report. Bethesda: National Foundation for Infectious Diseases; 2012.

[42] Services TUSDoHaH. Managing Asthma, A Guide for Schools. Bethesda: National Institutes of Health, National Heart, Lung, and Blood Institute; 1991 December 2014. Report No.: NIH Publication No. 14–2650.

[43] Gargano LM, Herbert NL, Painter JE, Sales JM, Vogt TM, Morfaw C, et al. Development, theoretical framework, and evaluation of a parent and teacher-delivered intervention on adolescent vaccination. Health Promot Pract. 2014;15(4):556–67. doi: 10.1177/1524839913518222 24440920 PMC5506681

[44] Barrett M, Gondalia R, Rowland C, Hill A, Attisha E, Kaye L, et al. Impact of a Digital Asthma Intervention on Short-acting Beta-agonist (SABA) Medication Use Among Medicaid-enrolled Children in Southwest Detroit. Journal of Allergy and Clinical Immunology. 2021;147(2):AB51.

[45] Lemanske RF Jr, Kakumanu S, Shanovich K, Antos N, Cloutier MM, Mazyck D, et al. Creation and implementation of SAMPRO™: A school-based asthma management program. J Allergy Clin Immunol. 2016;138(3):711–23. doi: 10.1016/j.jaci.2016.06.015 27596707 PMC5085063

[46] Liptzin DR, Gleason MC, Cicutto LC, Cleveland CL, Shocks DJ, White MK, et al. Developing, Implementing, and Evaluating a School-Centered Asthma Program: Step-Up Asthma Program. J Allergy Clin Immunol Pract. 2016;4(5):972–9.e1. doi: 10.1016/j.jaip.2016.04.016 27283054

[47] The School-Based Allergy, Asthma and Anaphylaxis Management Program™: Comprehensive Asthma Educational Resources Milwaukee: American Academy of Allergy, Asthma & Immunology; [Available from: https://www.aaaai.org/Tools-for-the-Public/School-Tools/SAMPRO.

[48] Seib K, Underwood NL, Gargano LM, Sales JM, Morfaw C, Weiss P, et al. Preexisting Chronic Health Conditions and Health Insurance Status Associated With Vaccine Receipt Among Adolescents. J Adolesc Health. 2016;58(2):148–53. doi: 10.1016/j.jadohealth.2015.10.009 26683985 PMC11898001

[49] Vlahov D, Bond KT, Jones KC, Ompad DC. Factors associated with differential uptake of seasonal influenza immunizations among underserved communities during the 2009–2010 influenza season. J Community Health. 2012;37(2):282–7. doi: 10.1007/s10900-011-9443-x 21785857 PMC3369692

[50] Cheung PT, Wiler JL, Lowe RA, Ginde AA. National study of barriers to timely primary care and emergency department utilization among Medicaid beneficiaries. Ann Emerg Med. 2012;60(1):4–10.e2. doi: 10.1016/j.annemergmed.2012.01.035 22418570

[51] Mudd AE, Michael YL, Diez-Roux AV, Maltenfort M, Moore K, Melly S, et al. Primary Care Accessibility Effects on Health Care Utilization Among Urban Children. Acad Pediatr. 2020;20(6):871–8. doi: 10.1016/j.acap.2020.05.014 32492576 PMC7261359

[52] Flores G, Olson L, Tomany-Korman SC. Racial and ethnic disparities in early childhood health and health care. Pediatrics. 2005;115(2):e183–93. doi: 10.1542/peds.2004-1474 15687426

[53] Solomon EM, Wing H, Steiner JF, Gottlieb LM. Impact of Transportation Interventions on Health Care Outcomes: A Systematic Review. Med Care. 2020;58(4):384–91. doi: 10.1097/MLR.0000000000001292 31985588

[54] Chiu APY, Dushoff J, Yu D, He D. Patterns of influenza vaccination coverage in the United States from 2009 to 2015. Int J Infect Dis. 2017;65:122–7. doi: 10.1016/j.ijid.2017.10.004 29042178

[55] Murphy PA, Frazee SG, Cantlin JP, Cohen E, Rosan JR, Harshburger DE. Pharmacy provision of influenza vaccinations in medically underserved communities. J Am Pharm Assoc (2003). 2012;52(1):67–70. doi: 10.1331/JAPhA.2012.10070 22257618

[56] Levy P, McGlynn E, Hill AB, Zhang L, Korzeniewski SJ, Foster B, et al. From pandemic response to portable population health: A formative evaluation of the Detroit mobile health unit program. PLoS One. 2021;16(11):e0256908. doi: 10.1371/journal.pone.0256908 34847164 PMC8631611

[57] Foradori DM, Sampayo EM, Fanny SA, Namireddy MK, Kumar AM, Lo HY. Improving Influenza Vaccination in Hospitalized Children With Asthma. Pediatrics. 2020;145(3). doi: 10.1542/peds.2019-1735 32107285

[58] Michigan CsHo. Immunization Station [Internet]. Detroit: TH Medical; 2022 [Available from: https://www.childrensdmc.org/services/immunization-station.

[59] Trump Administration Partners with Chain and Independent Community Pharmacies to Increase Access to Future COVID-19 Vaccines [Internet]. Washington, DC: The U.S. Department of Health & Human Services; 2020 [Available from: https://public3.pagefreezer.com/browse/HHS%20%E2%80%93%C2%A0About%20News/20-01-2021T12:29/https://www.hhs.gov/about/news/2020/11/12/trump-administration-partners-chain-independent-community-pharmacies-increase-access-future-covid-19-vaccines.html.

